# A New Paradigm for Training Hyperactive Dopamine Transporter Knockout Rats: Influence of Novel Stimuli on Object Recognition

**DOI:** 10.3389/fnbeh.2021.654469

**Published:** 2021-04-22

**Authors:** Natalia P. Kurzina, Anna B. Volnova, Irina Y. Aristova, Raul R. Gainetdinov

**Affiliations:** ^1^Institute of Translational Biomedicine, Saint Petersburg State University, Saint Petersburg, Russia; ^2^Department of Physiology, Faculty of Biology, Saint Petersburg State University, Saint Petersburg, Russia; ^3^Saint Petersburg State University Hospital, Saint Petersburg State University, Saint Petersburg, Russia

**Keywords:** dopamine transporter knockout rats, ADHD model, novelty, recognition memory, long-term memory

## Abstract

Attention deficit hyperactivity disorder (ADHD) is believed to be connected with a high level of hyperactivity caused by alterations of the control of dopaminergic transmission in the brain. The strain of hyperdopaminergic dopamine transporter knockout (DAT-KO) rats represents an optimal model for investigating ADHD-related pathological mechanisms. The goal of this work was to study the influence of the overactivated dopamine system in the brain on a motor cognitive task fulfillment. The DAT-KO rats were trained to learn an object recognition task and store it in long-term memory. We found that DAT-KO rats can learn to move an object and retrieve food from the rewarded familiar objects and not to move the non-rewarded novel objects. However, we observed that the time of task performance and the distances traveled were significantly increased in DAT-KO rats in comparison with wild-type controls. Both groups of rats explored the novel objects longer than the familiar cubes. However, unlike controls, DAT-KO rats explored novel objects significantly longer and with fewer errors, since they preferred not to move the non-rewarded novel objects. After a 3 months’ interval that followed the training period, they were able to retain the learned skills in memory and to efficiently retrieve them. The data obtained indicate that DAT-KO rats have a deficiency in learning the cognitive task, but their hyperactivity does not prevent the ability to learn a non-spatial cognitive task under the presentation of novel stimuli. The longer exploration of novel objects during training may ensure persistent learning of the task paradigm. These findings may serve as a base for developing new ADHD learning paradigms.

## Introduction

Attention deficit hyperactivity disorder (ADHD) is the most common disorder in children and adolescents (Posner et al., 2020; Shrestha et al., 2020). ADHD leads not only to poor attention but also to a decrease in motivated behavior and learning abilities (Volkow et al., 2011). In adulthood, certain ADHD symptoms persisted in up to 50% of cases. The patients are less hyperactive but have an impairment of working memory and selectivity of attention (Asherson et al., 2016; Luo et al., 2019). There are several approaches to ADHD diagnoses and treatment, but precise neurobiological mechanisms underlying ADHD pathology are insufficiently investigated so far. Generally, it is believed that ADHD is connected with the dopamine (DA) system dysfunction (Viggiano et al., 2004; Cinque et al., 2018). Beyond ADHD, DA dysregulation may contribute to numerous neurological and psychiatric disorders, such as Parkinson disease, Huntington’s disease, and schizophrenia (Russell et al., 2005; Tripp and Wickens, 2012; Ko et al., 2013; Ng et al., 2015; Grace, 2016; Creed and Goldberg, 2018; Konnova and Swanberg, 2018; Natsheh and Shiflett, 2018; Adinolfi et al., 2019). It is not surprising since the DA system is involved in the control of cognition, locomotion, reward evaluation, and formation of memories for reward–cue associations (Wise, 2004; Keiflin and Janak, 2015; Morrens et al., 2020).

It has been suggested that the development of ADHD and associated behavioral disorders might be caused by abnormalities in the functioning of the plasma membrane DA transporter (DAT). A significant association between ADHD and the gene encoding the DAT was demonstrated (Mill et al., 2005; Yang et al., 2007), and altered DAT expression in the striatum of patients with ADHD symptoms was shown (Madras et al., 2005). Furthermore, it is well known that patients with ADHD react favorably to therapy with Ritalin (methylphenidate) and Adderall (amphetamine), which target DAT (Dresel et al., 2000).

Dopamine transporter, by controlling re-uptake of released DA, plays a critical role in the regulation of both the intraneuronal and extracellular DA homeostasis (Vaughan and Foster, 2013). Mice lacking the DAT (DAT-KO mice) were shown to have high extracellular DA levels, which results in spontaneous hyperlocomotion and certain cognitive dysfunctions (Giros et al., 1996; Spielewoy et al., 2000; Gainetdinov et al., 2002; Efimova et al., 2016; Ide et al., 2018). In mutant mice, long-term alterations in DA signaling lead to profound disturbances in the cortico-striatal connections and functions (Surmeier et al., 2007). In general, numerous observations indicate that the DA system plays an important role in the consolidation of memory traces (Moncada and Viola, 2007; Beeler et al., 2010; Pezze and Bast, 2012; Duszkiewicz et al., 2019). Investigations in knockout mice allowed researchers to clarify many questions concerning the cognitive and behavioral processes that are critical for modeling neuropsychiatric disorders. The recent opportunity to translate these studies to transgenic rats significantly increased the capacity of such investigations. Rats are known to have a wider repertoire of behavioral reactions and can learn complex cognitive tasks (Abbott, 2004); thus, they are a more convenient model for investigating the cognitive functions.

The strain of rats with deletion of DAT gene (DAT-KO rats) was developed (Leo et al., 2018a) to investigate various aspects of DA system dysfunctions with a particular emphasis on cognitive disorders (Vengeliene et al., 2017; Leo et al., 2018b; Sukhanov et al., 2019). DAT-KO rats were described to have increased locomotor activity, an impaired sensorimotor gating, a deficit in operant nose-poke responding reinforced by food pellets, and decreased Y-maze spontaneous alternation. Furthermore, they have significant dysregulation in the fronto-striatal brain-derived neurotrophic factor function (Adinolfi et al., 2018; Cinque et al., 2018; Leo et al., 2018b). Recently, the impairment of spatial working memory was also demonstrated in DAT-KO rats. It was shown that they can learn the behavioral task in an eight-arm radial maze but perform it much less effectively than the control wild-type (WT) rats (Kurzina et al., 2020). Independently developed DAT deficient rats were also hyperactive, failed to show conditioned fear responses, were unable to learn stimulus–reward associations, and showed anhedonia and impaired cognition and social behaviors (Vengeliene et al., 2017; Sukhanov et al., 2019).

Novelty is a factor of the new environment that allows evaluation of the adaptation processes. Recognition memory is connected with the discrimination of novel information. In experiments in rodents, this type of memory is necessary to recognize previously encountered events or objects using combined information about stimuli and the environmental context where animals have encountered the objects including information about the location of the animal (Ergorul and Eichenbaum, 2004; Aggleton and Brown, 2006). Novel stimuli are known to excite DA neurons, which are involved in the exploratory activity in a novel environment (Costa et al., 2014). DAT-KO rats can recognize a novel testing chamber slightly different from the previous one (Adinolfi et al., 2018). Novel object recognition was found to depend on D2 DA receptor activity (França et al., 2016). In DAT knockdown mice, the impairment of novel object recognition was observed (Chang et al., 2020).

There are some new models to study long-term memory (Genzel et al., 2019) and new approaches for investigating memory consolidation (Hardt and Nadel, 2018) in normal rats, but DAT-KO rats were not evaluated yet in these tasks. So far, long-term memory in DAT-KO animals has not been investigated. Since memory improvement in ADHD patients was shown in experiments with novel stimulus presentation (Baumann et al., 2020), this approach is attracting attention to overcome ADHD problems without any medical treatment. The present study focuses on the evaluation of the ability of DAT-KO rats to learn the paradigm of object recognition with rewarded familiar objects and non-rewarded novel objects and store it in long-term memory.

## Materials and Methods

### Animals

Littermate male DAT-KO (*n* = 9) and control WT (*n* = 10) rats of 3–4 months’ age were used in the experiments. At the start of the experiments, body weight of DAT-KO rats was 274 ± 6 g and was lower than that of WT rats (292 ± 7 g). Rats were weighed daily. At the end of the experiments, body weight of DAT-KO rats was 250 ± 6 g, and in WT rats, body weight was 277 ± 8 g. The experiments were conducted in compliance with “The Regulations on Research Using Experimental Animals” (Order of Ministry of Health of USSR #742 of 13.11.1984), FELASA, and RusLASA requirements regarding the care and treatment of laboratory animals, and the Ethics Committee of Saint Petersburg State University, No. 131-03-4 of September 24, 2018. Before the experiments, rats were maintained in individually ventilated cages (IVCs) (RAIR IsoSystem World Cage 500) with unlimited access to food and water. The colony room was illuminated with artificial light from 9 a.m. to 9 p.m. All the experiments were conducted during the light portion of the light/dark cycle. For 5 days before the training, rats received a regular food ratio of 90% of the free-feeding amount (regular diet).

### Apparatus

**FIGURE 1:**
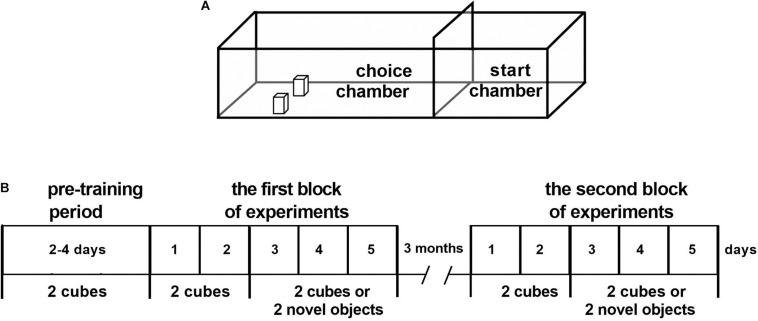
The construction of the apparatus **(A)** and the schedule of the experiments **(B)**. **(A)** Apparatus with the start and choice chambers. **(B)** Stages of experiments: the pre-training period (2–4 days); the first block of experiments: training with the rewarded objects (cubes, 2 days) and training with the rewarded cubes or non-rewarded novel objects (3 days); a 3-month interval; and the second block of experiments (5 days) using the same paradigm as during training.

The testing apparatus was arranged, and the experiments were performed as described (Gilbert and Kesner, 2003). The apparatus had the following characteristics: it consisted of a box with a 125 cm × 40 cm matte black floor and four 40-cm-high non-transparent red Plexiglas walls (Figure 1A). One removable red Plexiglas guillotine door (40 cm × 25 cm) was placed from one end of the box to divide the box into two separate compartments. The small (40 cm × 40 cm) compartment served as the start chamber, in which the rat began each trial, and the larger (85 cm × 40 cm) compartment served as the choice chamber in which the objects were presented. A 6 × 6 matrix of “food wells” (diameter, 2 cm; depth, 1 cm) was drilled in the floor at the end of the choice chamber. The rows and columns of food wells were separated by 2.5 cm.

### Task Procedure

#### Pre-training Period

The pre-training period included handling and animal’s familiarization with the test apparatus (Figure 1B). Before the experiments, during 2 days, each animal was handled for 20 min daily and was allowed to individually explore the test apparatus for 0.30 h.

The experiment aimed at training rats to solve a behavioral task. Ten pieces of popcorn breakfast loops (produced by Nestle S.A.) were spread out on the floor surface of the choice chamber. Following 2 days for WT rats and 4 days for DAT-KO rats, two objects were introduced into the testing apparatus. Two wooden cubes of the same weight and dimensions (5 cm × 4 cm × 4 cm) painted white were used as objects. They were placed to cover the center of the two food wells in the choice chamber of the apparatus. The cubes were placed on the two food wells (third row, left and right sides, Figure 1A). Rats were trained to move objects to obtain food reinforcement. On each training trial, a piece of reward was put in front of the objects on the maze floor surface. The animal was placed in the start chamber, with a guillotine door between the start and choice chambers in the closed position. The animal was allowed to exit the start chamber, retrieve a reward from the choice chamber, and return to the start chamber to consume the reward with the door in the closed position. Some animals preferred to consume food reward near the object. Once the animal retrieved the food reward consistently on the first day, the food reward was placed in the food well, which was previously covered by the object, and the object was positioned on the side of the food well opposite the animal. On each ensuing trial, the object was positioned to cover a larger portion of the food well, until the base of the object covered the baited food well completely. After that, we started the experiments *per se*.

#### First Block of Experiments (Training Period)

During two consecutive days (days 1 and 2, Figure 1B), two cubes were presented 10 times daily until rats were able to displace them from the completely closed food wells. During the following 3 days, the cubes and novel objects were presented 16 times in a semi-random manner (in random manner, but not more than three times consecutively). The cubes were presented 11 times and novel objects five times. The novel objects were never repeated and never contained any food reward under them. Thus, the first block of the experiments for obtaining data for statistical analysis lasted for 5 days: 2-day training sessions with only the cubes and 3-day training sessions with the cubes or novel objects (Figure 1B).

#### The Second Block of Experiments

After a 3-month interval, the second block of the experiments was conducted to check the storage of elaborated motor skills in the rats’ long-term memory. Without any pre-training, rats were retested for 5 days using the same training paradigm as in the first experimental block: for 2 days when only the cubes were presented and for 3 days with the cubes or novel objects presented semi-randomly.

All behavioral parameters were calculated by EthoVision XT video tracking system (Noldus Information Technology, Netherlands). In both blocks of the experiments, the following behavioral parameters were measured: (1) cumulative duration of task fulfillment (the level of locomotor activity); (2) cumulative distances covered by rats during task performance (hyperactive and perseverative patterns of activity); (3) the latent period of the rat running to the first object (the object recognition); (4) cumulative duration of the object explorations, cubes or novel objects (curiosity level, recognition, and remembering the objects’ shape); and (5) number of erroneous running – rats displacing the non-rewarded novel objects or not displacing the rewarded ones (erroneous recollection of the paradigm rules).

### Statistical Analysis

The data were presented as mean ± SEM; *p* < 0.05 was considered statistically significant for all tests. Preliminary estimation of data distribution normality (Gaussian distribution) was done using the Kolmogorov–Smirnov test. Differences between the mean values of registered parameters were estimated by the two-tailed unpaired Mann–Whitney test for two-group comparison. Differences across group mean values were tested for significance with one-way ANOVA for the data with normal distribution and with one-way analysis of variance by the Kruskal–Wallis test for the data with the non-normal distribution. Dunn’s multiple-comparison *post hoc* test was also used. All calculations were performed in GraphPad Prism 7 (GraphPad Software, Inc., San Diego, CA, United States).

## Results

### The First Block of Experiments

We found that both groups of rats were able to learn to move the cubes and retrieve a food reward. However, it should be noted that the pre-training period was longer in DAT-KO rats (4 days) in comparison with WT rats (2 days). At the end of the pre-training, all rats were able to correctly perform the behavioral task of moving the cubes and retrieving a reward from the food wells.

**FIGURE 2:**
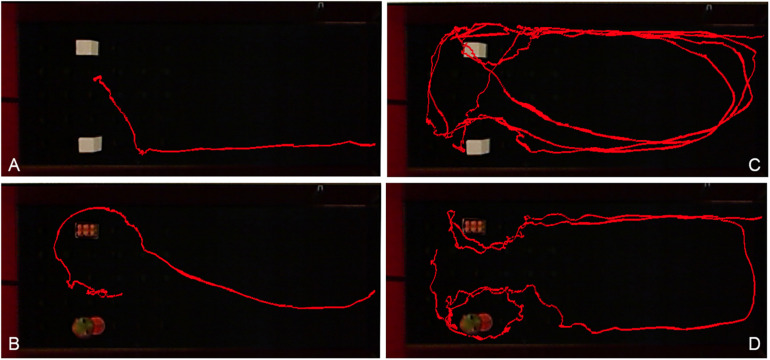
Comparison of visual tracking examples of WT **(A,B)** and DAT-KO rats **(C,D)** during training with cubes **(A,C)** or novel objects **(B,D)**. WT, wild-type; DAT-KO, dopamine transporter knockout.

The analysis of video tracking showed evident differences between the two groups (Figure 2). The WT rats ran directly to the object (Figures 2A,B) and immediately retrieved a reward, while DAT-KO rats’ trajectory was more complex; they explored the objects longer before retrieving food (Figures 2C,D). They would run in a perseverative manner around the arena before stopping near the object.

We did not find any differences in all behavioral parameters measured when the rewarded objects (cubes) were presented alone or alternating with novel objects. Due to it, our data regarding the cubes are summarized for all the parameters analyzed without any differentiation as to how they were presented.

**FIGURE 3:**
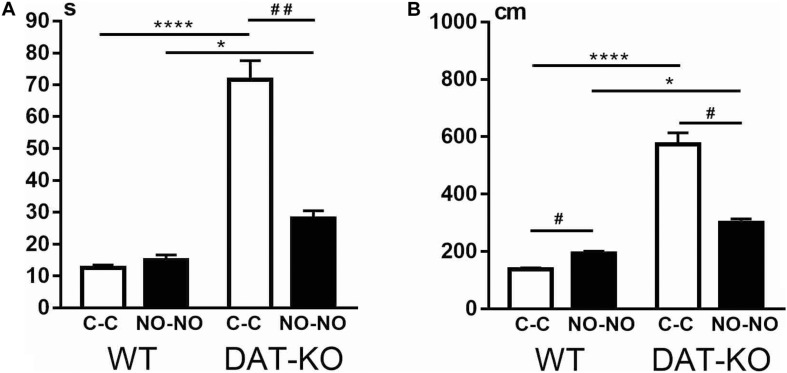
Comparison of task fulfillment duration in seconds **(A)** and distances covered in centimeters **(B)** in WT and DAT-KO rats during the presentation of cubes (C-C, white columns) and novel objects (NO-NO, black columns); * or ^#^*p* < 0.05; ^##^*p* < 0.01; ****p < 0.0001; one-way Kruskal–Wallis test with Dunn’s multiple-comparison *post hoc* test. WT, wild-type; DAT-KO, dopamine transporter knockout.

The analysis of the locomotion level by the cumulative duration of task performance showed that there are significant differences between WT and DAT-KO rats: DAT-KO rats spent a longer time on task fulfillment (Figure 3A). The one-way analysis of variance by the Kruskal–Wallis test with Dunn’s multiple-comparison test showed that DAT-KO rats spent longer time not only to displace the cubes (*p* < 0.0001) but also to explore the novel objects (*p* < 0.05). Analysis of the time course of the task fulfillment revealed that DAT-KO rats had a shorter duration of running to the novel objects (*p* < 0.01) than to the cubes, whereas no differences in duration of time during the cube and novel object presentations were found in WT rats.

The hyperlocomotion of DAT-KO rats was also evident when distance covered during task fulfillment was measured (Figure 3B). Thus, in comparison with WT rats, DAT-KO rats were observed to cover significantly longer distances during the cube (*p* < 0.0001) and novel object (*p* < 0.05) presentations. In contrast, WT rats traveled longer distances when novel objects but not cubes were presented (*p* < 0.05). On the contrary, DAT-KO rats traveled (*p* < 0.05) shorter distances when novel objects were presented.

**FIGURE 4:**
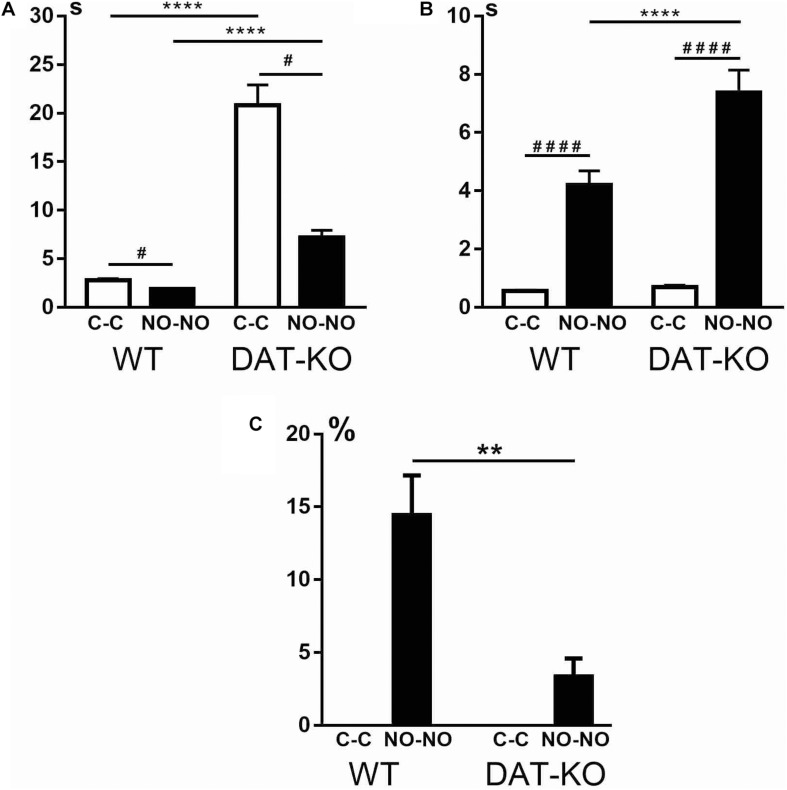
Comparison of latent periods of running to the first object **(A)**, time of object exploration **(B)**, and the number of erroneous trials **(C)** in WT and DAT-KO rats during presentation of cubes (C-C, white columns) and novel objects (NO-NO, black columns); ^#^*p* < 0.05; **p < 0.01; **** or ^####^*p* < 0.0001; one-way Kruskal–Wallis test combined with Dunn’s multiple-comparison *post hoc* test. WT, wild-type; DAT-KO, dopamine transporter knockout.

The ability of animals to recognize objects was evaluated by detecting latent periods of running to the first object (Figure 4A). It was observed that DAT-KO rats spent significantly longer periods of their time to reach the first object (the cubes or novel objects) than controls (the one-way analysis of variance by Kruskal–Wallis test with Dunn’s multiple-comparison *post hoc* test, *p* < 0.0001). It should be noted that both groups of rats demonstrated a decrease in time when reaching the novel objects in contrast to the cubes (*p* < 0.05).

In recognition memory tasks, evaluation of the duration of object exploration is very important. The difference in time of exploration between novel and familiar objects reflects their ability to recognize objects. The curiosity level and recognition of objects of both groups of rats were evaluated by detection of the time of object exploration. Both groups of rats showed (Figure 4B) that exploration of novel objects took significantly longer time than that of familiar objects (one-way analysis of variance by Kruskal–Wallis test with Dunn’s multiple-comparison *post hoc* test, *p* < 0.0001). Duration of the novel object exploration was found to be longer in DAT-KO rats (*p* < 0.0001) than in controls, whereas no significant changes were revealed in the duration of exploring familiar objects (Figure 4B).

Both groups of rats perfectly recognized familiar objects (cubes), and the level of correct running was 100% (Figure 4C). The errors in recognizing objects when rats moved the non-rewarded novel objects showed that DAT-KO rats made significantly fewer errors than WT rats (two-tailed Mann–Whitney test, *p* < 0.01).

Taken together, these data suggest that longer distances covered by DAT-KO rats during the object presentation and longer period of time of the task fulfillment reflect their hyperlocomotion and a high level of perseverative activity. The longer time that DAT-KO rats took in the latent periods running to the first object in order to move it suggests that they recognize the objects slower than do WT rats. On the other hand, DAT-KO rats can better memorize the reward absence related to the novel objects, likely due to longer exploration, and subsequently, with less incorrect choices.

### The Second Block of Experiments – Retesting of Wild-Type and Dopamine Transporter Knockout Rats After a 3-Month Interval

In all the figures below, significant differences are marked only for the comparison between the first and second blocks of the experiments. When retested, DAT-KO and control rats were capable of performing the task starting from the first day of the experiments. Both groups demonstrated a 100% level of correct trials in the paradigm when only the cubes were presented. To reveal the retention of the learned skills, the results for the first and second blocks of the experiments were analyzed across both groups of rats.

**FIGURE 5:**
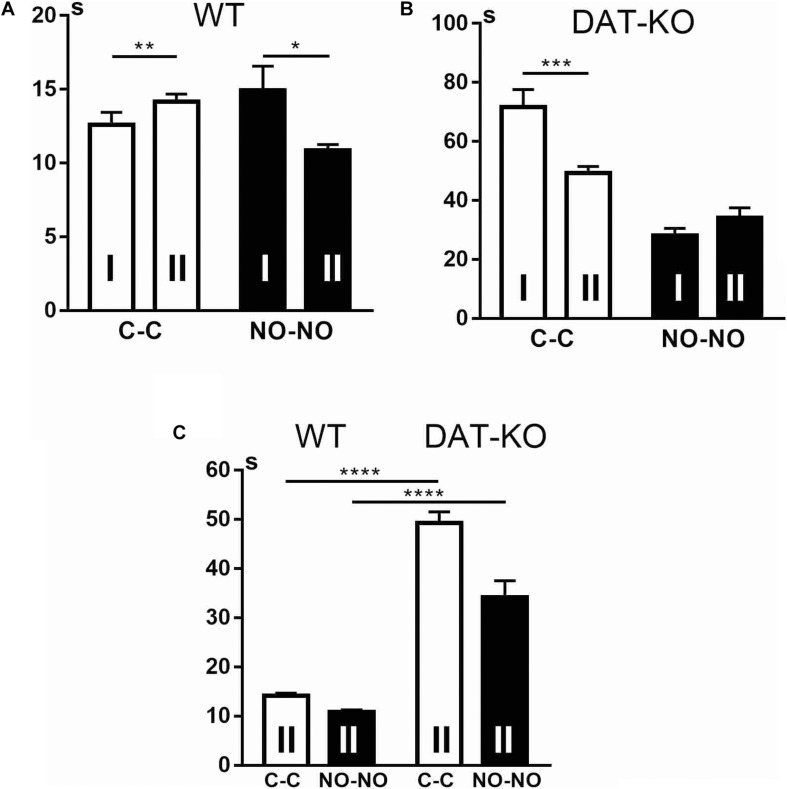
Comparison of task fulfillment duration in WT **(A,C)** and DAT-KO rats **(B,C)** during the first (I) and second (II) blocks of experiments. White columns, cube presentation (C-C); black columns, novel object presentation (NO-NO); *p < 0.05; **p < 0.01; ***p < 0.001; ****p < 0.0001; one-way Kruskal–Wallis test with Dunn’s multiple-comparison *post hoc* test. WT, wild-type; DAT-KO, dopamine transporter knockout.

Remembering of the task conditions occurred in both groups but in different ways. The WT rats remembered task rules very well and even showed some exploratory activity reflected in the significant increase (*p* < 0.01) of the time spent in the arena when the cubes were presented (Figure 5A) and decrease when novel objects were presented (*p* < 0.05). The DAT-KO rats also remembered the task conditions, but it was reflected (Figure 5B) in decrease in hyperlocomotion and perseverative activity. DAT-KO rats spent significantly (*p* < 0.001) less time in the arena during the cube presentations than in the first block of the experiments, whereas the time for the novel object presentations did not differ significantly. Cumulative duration of task fulfillment, when compared in WT and DAT-KO rats (Figure 5C), shows that as in the first block of the experiments, in the second block, WT rats performed the task significantly (*p* < 0.0001) faster than DAT-KO rats during cube and novel object presentations.

**FIGURE 6:**
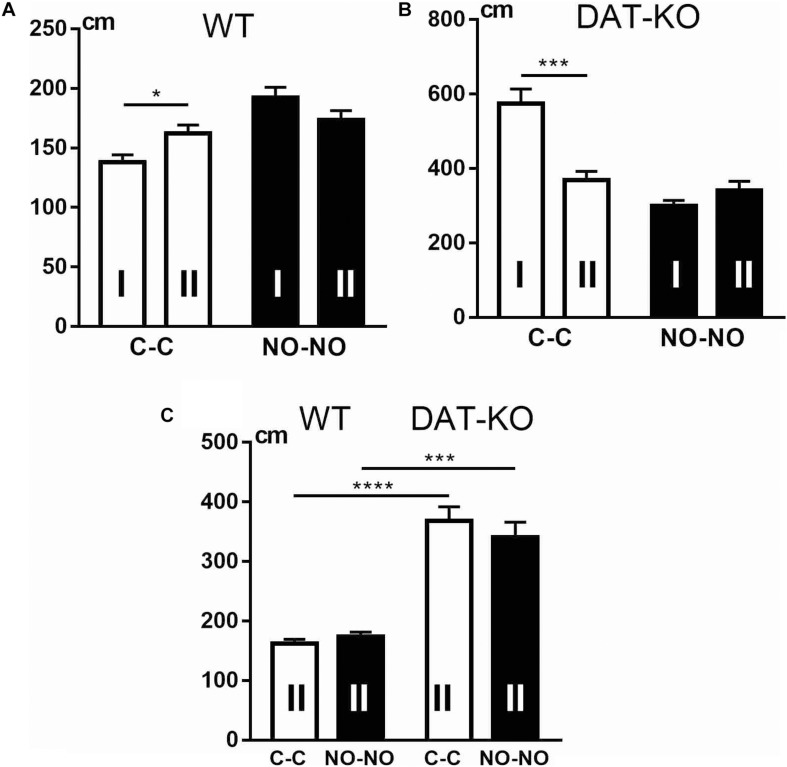
Comparison of distances covered by WT **(A,C)** and DAT-KO rats **(B,C)** in the first (I) and second (II) blocks of experiments. White columns, cube presentations (C-C); black columns, novel object presentations (NO-NO); *p < 0.05; ***p < 0.001; ****p < 0.0001; one-way Kruskal–Wallis test with Dunn’s multiple-comparison *post hoc* test. WT, wild-type; DAT-KO, dopamine transporter knockout.

We compared the distances covered by WT and DAT-KO rats during the cube presentation in the first and second blocks of the experiments (Figure 6) and found that WT rats (Figure 6A) covered longer distances (*p* < 0.05) in the second block of the experiments than during the first. In contrast, in the second block of the experiments, DAT-KO rats (Figure 6B) covered shorter distances during the cube presentation (*p* < 0.001). None of the groups revealed any significant differences in the distances traveled during the novel object presentations (Figures 6A,B). As to the distances covered by both groups, WT rats ran significantly shorter distances than DAT-KO rats (Figure 6C) during the presentations of the familiar (*p* < 0.0001) and novel (*p* < 0.001) objects.

**FIGURE 7:**
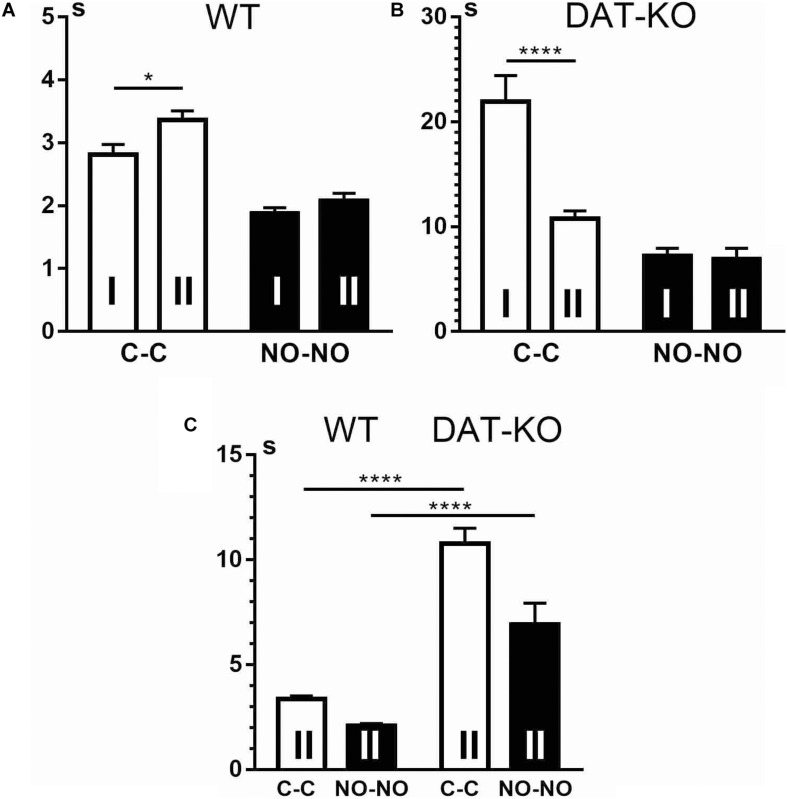
Comparison of the latent periods of running to the first object in WT **(A,C)** and DAT-KO rats **(B,C)** in the first (I) and second (II) blocks of experiments. White columns, cube presentation (C-C); black columns, novel object presentation (NO-NO); *p < 0.05; ****p 0< 0.0001; one-way Kruskal–Wallis test with Dunn’s multiple-comparison *post hoc* test. WT, wild-type; DAT-KO, dopamine transporter knockout.

We found that during the second block of the experiments, WT rats (Figure 7A) showed an increase in latent periods of running to the first object to move it (cubes) (*p* < 0.05). In contrast, in DAT-KO rats (Figure 7B), time of running to the cubes decreased significantly (*p* < 0.001). Neither of the two groups revealed any significant differences in time of running to the novel objects (Figures 7A,B). Latent periods measured for running to the first object showed (Figure 7C) that as in the first block of the experiments, in the second block, WT rats ran significantly faster than DAT-KO rats (*p* < 0.0001).

**FIGURE 8:**
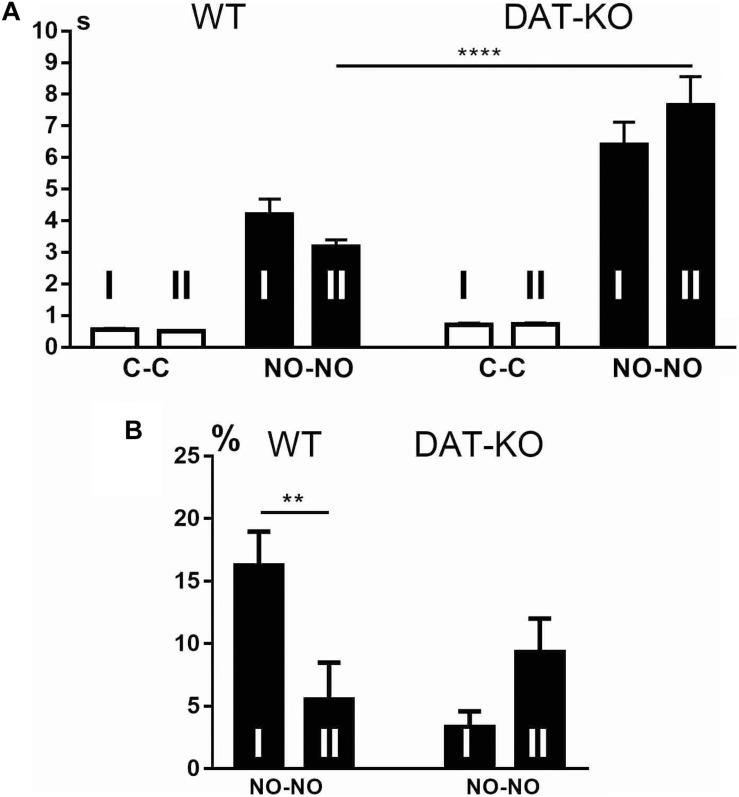
Comparison of the time of object exploration by WT and DAT-KO rats **(A)**, and the number of erroneous trials in WT and DAT-KO rats during novel object presentations **(B)** in the first (I) and second (II) blocks of experiments. White columns, cube presentation (C-C); black columns, novel object presentations (NO-NO); **p < 0.01; ****p < 0.0001; one-way Kruskal–Wallis test with Dunn’s multiple-comparison *post hoc* test. WT, wild-type; DAT-KO, dopamine transporter knockout.

Similar to the first experimental block, during the second block, WT and DAT-KO rats explored novel objects (*p* < 0.0001) longer (Figure 8A) than cubes. We failed to find any significant differences in the time of the familiar object explorations in both groups during the first and second blocks of the experiments (Figure 8A). The analysis of the duration of the novel object exploration showed that DAT-KO rats spent significantly (*p* < 0.0001) longer periods of their time than did WT rats when exploring novel objects (Figure 8A).

In the first and second blocks of the experiments, both groups of rats showed no erroneous trials during the cube presentations. The comparison of the erroneous trials during the novel object presentations (Figure 8B) showed significantly better performance in control rats (*p* < 0.01) during the second block of the experiments, whereas no significant changes were found in DAT-KO rats. The comparison of the number of erroneous trials during the second block of the experiments showed no significant differences between both groups (Figure 8B) when they moved the non-rewarded novel objects.

Taken together, the data from the second block of the experiments suggest that DAT-KO rats not only learned non-spatial behavioral task rules but stored them in memory. From the very beginning of re-testing, DAT-KO animals, like controls, were capable of moving the cubes correctly. Moreover, DAT-KO rats demonstrated good remembering of the task conditions, as reflected by the fact that the time they needed to run and move the first object was shorter.

## Discussion

Here, we present the data obtained in the experiments with DAT-KO rats that display behavioral abnormalities relevant to ADHD endophenotypes. These rats have elevated extracellular DA levels and spontaneous hyperactivity. We investigated the influence of the task paradigm on the learning and storage in the memory motor skills by DAT-KO rats. Despite the difficulties with task acquisition, they have demonstrated an ability to learn a novel object recognition task and store the learned paradigm in memory for 3 months.

Hyperactivity of DAT-KO rats and mice and perseverative pattern of motor activity was demonstrated in numerous investigations and are described as their distinctive traits (Pogorelov et al., 2005; Vengeliene et al., 2017; Adinolfi et al., 2018; Cinque et al., 2018; Ide et al., 2018; Leo et al., 2018b). DAT-KO rats spent much longer time and covered longer distances to perform a task, which might be due to their hyperactivity. DAT-KO rats also spent a longer time to reach the first target to be moved. It might be suggested that their object recognition abilities are inferior and not as good as those of WT rats. This fact might be also explained by their hyperactivity and perseverations. The inability of DAT-KO rats to immediately focus their attention on the objects presented may be also related to their attention deficit described earlier (Sukhanov et al., 2019).

In our previous study, we found that DAT-KO rats can learn the spatial cognitive task in the eight-arm radial maze, but the task fulfillment was significantly worse than in control rats. In the spatial task, DAT-KO rats also spent significantly more time and covered longer distances during training than WT rats. DAT-KO rats also demonstrated an increased perseverative pattern during the spatial task. Analysis of repeated entry sequences showed that DAT-KO rats had a sustainable statistically significant tendency to increase the arms revisits (Kurzina et al., 2020). Highly perseverative patterns of locomotor activity during eight-arm radial maze task were previously reported in DAT-KO mice (Gainetdinov et al., 2002; Efimova et al., 2016).

The recognition memory processes, underlying the ability to choose between the stimuli that have occurred previously and the novel ones, are fundamental for recording events and controlling the target of perspective behaviors. Recognition memory is based on various types of information that allows establishing whether a stimulus has been previously encountered and how it might affect subsequent behaviors (Kinnavane et al., 2015). In a place recognition test with a long (24 h) delay period, a 20-min familiarization was sufficient for rats to discriminate between novel and familiar objects (Ozawa et al., 2011). In associative recognition memory tests, rats would make the association between objects, places, and contexts (Antunes and Biala, 2012). The significant differences in time of the novel and familiar object exploration reflect the ability of animals to react to novelty factors. The main difference in our training paradigm is in using unrewarded novel objects and rewarded familiar ones. As a rule, no reward is used in the recognition memory tasks. We found that DAT-KO rats spent long periods of their time to explore novel objects than WT rats. It is known that blocking the DAT leads to an increased preference for novel options in probabilistic decisions: for instance, DAT blockade in monkeys does not modulate the rate of the task learning (Costa et al., 2014). Hyperdopaminergic DAT-KO mice are easily aroused by novelty and always respond with a hyperlocomotion (Spielewoy et al., 2000). We believe that prolonged exploration observed in our task provides an opportunity for better encoding the distinctions between novel and familiar objects.

During learning, DAT-KO rats were making fewer erroneous trials than were WT rats. It might indicate that protracted novel object exploration leads to the imprinting in memory the learned rule that a novel object has never been rewarded. In the present study, the task had two aspects – motor reactions and object recognition. The rats had to run to retrieve food and at the same time to differentiate the objects to be moved. It should be pointed out again that unlike in other recognition tasks, rats received food reinforcement that helped them to memorize the task rules. The task specificity likely affects the DAT-KO rats’ reactions to the rewarded objects. However, in earlier studies that employed other tasks, it was found that DAT-KO rats are less sensitive to the rewarded stimuli than WT rats (Cinque et al., 2018). Some investigations describe the reward deficiency syndrome, which includes ADHD as a behavioral subtype. The patients with dysfunction of the brain reward cascade in the DA system have a high risk of addictive and compulsive behaviors (Blum et al., 2008). It is possible that the dysfunction of the brain reward system may also exist in DAT-KO rats. When we use an attractive reward, we improve the reward system functioning. Thus, reward may play the role of a specific trigger for storing the information of the experimental paradigm in long-term memory. Our experimental paradigm included the new objects that were never repeated, and therefore, the rat’s curiosity and motivation level might have increased. Moreover, regular rewarding of the familiar objects also contributes to task learning.

Change in DA neurotransmission does not necessarily result in alterations of motor skill acquisition and novel stimulus discrimination. Investigations in DAT knockdown (DAT-KD) mice showed that the elevated DA level did not affect instrumental learning but reduced the selectivity of the stimulus control in the Pavlovian conditional training (Yin et al., 2006). In our studies, DAT-KO rats learn and perform motor tasks easier than spatial tasks (Kurzina et al., 2020).

Examining long-term storage of motor skills in the conditions of the novel object presentations, we found that after 3 months, both groups of rats were able to correctly perform behavioral tasks starting from the first day of the retesting. A comparison of almost all behavioral parameters measured showed that DAT-KO rats performed the task better than WT rats. It was only the number of erroneous trials that did not vary significantly across the groups. There are data to indicate that the extension of a period of familiarization with the objects improved the long-term complex associative recognition memory (Shimoda et al., 2019). Acquisition of the task rules in both groups of rats may likely proceed similarly and thus help memory consolidation.

The most interesting findings were obtained when the results of the first and second experimental blocks were compared. DAT-KO rats have improved their results significantly – they performed the task faster, covered shorter distances, and spent shorter periods of their time to reach the first object when familiar objects were presented. We believe that motor task in a stable environment with an additional stimulus with rewarded objects contributes to a task acquisition for a long period. This task based on learning the motor skills is closely connected with the cortico-basal ganglia and the ventral tegmental area (VTA)–hippocampus connections (Hosp et al., 2009). Long-term potentiation (LTP) is one of the cellular mechanisms of long-term memory. It was shown that LTP/long-term depression (LTD) in the rat prefrontal cortex is modulated by DA (Otani et al., 2015), and altered cortical and nucleus accumbens LTP is found in DAT-KO mice (Yao et al., 2004; Xu et al., 2009). Rats that were placed into a novel environment that resembled an environment that they had previously encountered showed activation of D1/D5 DA receptors and synthesis of plasticity-related proteins leading to formation of long-term memory traces (Moncada and Viola, 2007). Likely, novel experiences that have some features in common with those of the past may activate the DA system and help memory consolidation (Duszkiewicz et al., 2019). In our case, an increased DA signaling may also lead to a better consolidation of memory traces.

In general, the data obtained allow us to suggest that DAT-KO rats learn and perform motor tasks easier in comparison with spatial tasks. We have found previously that DAT-KO rats are unable to form an optimal strategy for food search in comparison with WT rats. They also have a pronounced impairment in working memory (Kurzina et al., 2020). Intriguingly, in children with ADHD, the impairment of working memory components related to attention was described (Rodríguez-Martínez et al., 2020). At the same time, children with ADHD have intact long-term memory but a mild deficit in visual-spatial memory (Kibby and Cohen, 2008).

Furthermore, since DA is involved in various aspects of motivational behaviors and psychotic states (Efimova et al., 2016; Howe et al., 2018), the abnormal behaviors of DAT-KO animals may have relevance also for endophenotypes of other psychiatric disorders such as schizophrenia, bipolar disorder, autism, mania, obsessive–compulsive disorder, and addiction disorders (Arime et al., 2012; Wong et al., 2012; Jones et al., 2015; Mereu et al., 2017; Weinstein et al., 2017; Chang et al., 2018; Cinque et al., 2018; Chao et al., 2020). The DAT-KO rats might have good translational value for developing new treatment principles for mental disorders. As regards ADHD, it was reported that in children and adolescents with ADHD, exploration of a novel environment led to significantly better memory consolidation (Baumann et al., 2020). Data indicate that cognitive training of executive functions is more effective in preschooler children with symptoms of ADHD than in children without any developmental risks (Scionti et al., 2020). Children with ADHD were trained to fulfill a working memory task with individual feedback; and a year after training, they showed a pronounced improvement of working memory (Dobrakowski and Łebecka, 2020). Our studies in the animal model based on a goal-directed task may suggest development of new techniques improving learning in hyperactive children.

## Conclusion

The data obtained indicate that DAT-KO rats can learn and retrieve the object recognition task from memory following a 3-month interval. Their task performance differs from that of WT rats, as reflected by a longer time of task fulfillment and the distances covered. Also, in a novel object exploration, DAT-KO rats took a longer time. However, they made fewer erroneous trials than WT rats. After a 3-month interval, both DAT-KO and WT rats correctly performed the behavioral task. During the retesting, DAT-KO rats showed remarkable progress: they fulfilled the task faster and traveled shorter distances when the familiar objects were presented than during learning. It means that under the condition of the novel object presentation, hyperactive DAT-KO rats can learn and store motor tasks in memory. Consequently, the data obtained suggest the development of innovative strategies for teaching children with ADHD. A novel stimulus included in the training material may improve the quality of learning. Switching from the routine materials to something new is likely to provide the basis for better information consolidation.

## Data Availability Statement

The raw data supporting the conclusions of this article will be made available by the authors, without undue reservation.

## Ethics Statement

The animal study was reviewed and approved by the Ethics Committee of Saint Petersburg State University, No. 131-03-4 of September 24, 2018.

## Author Contributions

NK, AV, and IA performed the experiments and data analysis. NK, AV, and RG designed the study and contributed to the writing and editing of the manuscript. RG contributed to the conceptualization, supervision, and funding acquisition for the project. All authors contributed to the article and approved the submitted version.

## Conflict of Interest

The authors declare that the research was conducted in the absence of any commercial or financial relationships that could be construed as a potential conflict of interest.
